# Access, acceptability, and uptake of the COVID-19 vaccine among global migrants: A rapid review

**DOI:** 10.1371/journal.pone.0287884

**Published:** 2023-06-30

**Authors:** Higinio Fernández-Sánchez, Ziad Zahoui, Jennifer Jones, Emmanuel Akwasi Marfo

**Affiliations:** 1 Research Department, Cizik School of Nursing, University of Texas Health Science Center at Houston, Houston, Texas, United States of America; 2 Faculty of Nursing, University of Alberta, Edmonton, Alberta, Canada; University of Buea, CAMEROON

## Abstract

**Objective:**

To conduct a rapid review and determine the acceptability, access, and uptake of the COVID-19 vaccine among global migrants.

**Materials and methods:**

A rapid review was conducted May 2022 capturing data collected from April 2020 to May 2022. Eight databases were searched: PubMed, Ovid Medline, EMBase, CINAHL, SCOPUS, Google Scholar, LILACS, and the Web of Science. The keywords “migrants” AND COVID-19” AND “vaccine” were matched with terms in MeSH. Peer-reviewed articles in English, French, Portuguese, or French were included if they focused on COVID-19 immunization acceptability, access, or uptake among global migrants. Two independent reviewers selected and extracted data. Extracted data was synthesized in a table of key characteristics and summarized using descriptive statistics.

**Results:**

The search returned 1,186 articles. Ten articles met inclusion criteria. All authors reported data on the acceptability of the COVID-19 vaccine, two on access, and one on uptake. Eight articles used quantitative designs and two studies were qualitative. Overall, global migrants had low acceptability and uptake, and faced challenges in accessing the COVID-19 vaccine, including technological issues.

**Conclusions:**

This rapid review provides a global overview of the access, acceptability, and uptake of the COVID-19 vaccine among global migrants. Recommendations for practice, policy, and future research to increase access, acceptability, and uptake of vaccinations are discussed.

## Introduction

On March 11, 2020, the World Health Organization (WHO) [1] categorized COVID-19 a pandemic prompting an accelerated timeline for development and distribution of an effective vaccine. By December 2020, countries began mass vaccination programs starting with essential workers, vulnerable groups, and older adults [2]. As effective vaccines have become available to a wide range of populations, the WHO acknowledged migrants as a group that may be at greater risk of experiencing the burdens of the COVID-19 pandemic [3].

Global migrants include individuals voluntarily or involuntarily living outside of their birth country. The 2020 World Migration Report [4] estimated 164 million migrant workers globally in 2017. Migrants are among the populations that are at greater risk for structural discrimination and vulnerability, conditions that have been aggravated during the COVID-19 pandemic due to the restriction in access to universal rights, such as health, food, housing, and work [5]. Because global migrants face precarious conditions and systemic health inequities, [6, 7] COVID-19 disproportionately affects them [8, 9] and there is a need to understand their access and uptake of COVID-19 vaccines.

The International Organization for Migration reported that 83% of migrants in documented situations had access to COVID-19 vaccines, whereas only 46% of migrants with undocumented status have access to the vaccine, compiling data from 180 countries [10]. This is congruent with the literature, where undocumented migrants tend to be at a greater disadvantage when it comes to accessing health care while still holding essential jobs [10–12]. To the best of our knowledge, no published article synthesizes the literature on COVID-19 vaccine access, acceptability, and uptake among global migrants. Knowledge about COVID-19 vaccine access, acceptability, and uptake will inform policymaking and interventions to alleviate the negative impact of COVID-19 on global migrants. A vaccine continuum encompassing five factors can help with identifying the precursors to vaccine uptake: awareness, availability, accessibility, affordability, and acceptability [13].

To determine the acceptability, access, and uptake of the COVID-19 vaccine among global migrants, we conducted a rapid literature review to locate the literature on COVID-19 vaccine access, acceptability, and uptake among global migrants amid the pandemic.

## Materials and methods

### Design

We conducted a rapid review of the acceptability, access, and uptake of the COVID-19 vaccine among global migrants published between 2020–2022. This review followed the guidelines that the World Health Organization [14] and the Cochrane Collaborations [15] proposed. This approach is appropriate when urgent and surfacing health questions need to be answered in a timely manner, such as concerns related to the current COVID-19 pandemic. The review question was developed following the population-concept-context (PPC) framework proposed by the Joanna Briggs Institute to identify the main concepts in review questions [16]. The utilization of the PCC framework (population, concept, and context) is advised as a valuable tool for formulating explicit and meaningful objectives, as well as establishing eligibility criteria, in the context of a scoping review. The population was global migrants, and the concept was COVID-19 vaccine acceptability, access, and uptake in the contexts of the COVID-19 pandemic and vaccination [16].

### Eligibility criteria

For this study, specific inclusion criteria were established to determine which reports would be considered. Firstly, reports were eligible if they addressed the topics of COVID-19 immunization acceptability, access, or uptake among global migrants. This ensured that the focus of the research was directly relevant to the intended investigation. Secondly, only peer-reviewed articles published in academic journals were included. This criterion aimed to ensure that the included reports underwent a rigorous evaluation process by experts in the field, enhancing the credibility and reliability of the findings. Furthermore, the study encompassed reports employing various research methodologies, including quantitative, qualitative, or mixed-methods designs. This broad approach allowed for a comprehensive exploration of the research topic, incorporating diverse perspectives, and capturing both numerical data and qualitative insights. Lastly, reports written in either English or any of the Romance languages, such as Spanish, Portuguese, and French, were considered eligible for inclusion. By including reports in these languages, the study aimed to encompass a broader range of literature and promote inclusivity across different linguistic communities. Overall, the application of these inclusion criteria ensured a focused and comprehensive analysis of reports on COVID-19 immunization among global migrants, while also considering various research methodologies and linguistic diversity.

We excluded articles that were not research studies (i.e., commentaries, editorials); articles that did not segregate migrants’ data from other populations; articles that did not speak about the acceptability, access, or uptake of vaccines other than the COVID-19 vaccine; and the grey literature.

### Data sources and literature search

The lead author, HFS, conducted the database search on May 1, 2022. Eight databases were searched: Ovid Medline, EMBASE, CINAHL, SCOPUS, Google Scholar, and the Web of Science Core Collection. The keywords (migrants, COVID-19, and vaccine-COVID-19 pandemic) were matched with terms in MeSH. The search was conducted using a combination of the subject headings and keywords shown in Table 1; the search strategy was tailored for each database.

**Table 1:** Keywords used to search main databases.

**AND**			
**OR**	Migrant* OR Transient* OR Migrant worker* OR Nomad* OR Refugee* OR Immigrant*	Covid-19 vaccine* OR Covid-19 virus vaccine* OR SARS-CoV-2 vaccine* OR SARS2 vaccine* OR Coronavirus disease 2019 vaccine* OR Coronavirus disease-19 vaccine* OR 2019-nCoV vaccine* OR 2019 nCoV vaccine* OR SARS coronavirus 2 vaccine	COVID 19 OR SARS-CoV-2 Infection OR 2019 Novel Coronavirus Disease* OR 2019 Novel Coronavirus Infection* OR 2019-nCoV Disease* OR COVID-19 Virus Infection* OR Coronavirus Disease 2019 OR Severe Acute Respiratory Syndrome Coronavirus 2 Infection* OR SARS Coronavirus 2 Infection* OR 2019-nCoV Infection* OR COVID-19 Pandemic

### Study screening and selection

Two independent reviewers, HFS and ZZ, screened and selected the articles for their titles and abstracts; remaining records were screened and selected as full texts. A third reviewer, JJ, or EM, screened all the excluded full-text articles. Conflicts that arose during the review were resolved via general agreement among the reviewers. We used the Covidence TM 2.0 systematic review software to assist in the review process [17]. In particular, the software helped detect duplicates, store articles, and it allowed the reviewers to independently review and select articles.

### Data extraction and knowledge synthesis

One reviewer, ZZ, extracted the research data, and a second reviewer, HFS, verified the data for accuracy and comprehensiveness. A structured summary table that the research team designed was piloted and used to extract relevant data from the articles. The data extracted is available in Tables 1 and 2. A narrative summary approach was utilized to synthesize the data extracted. Additionally, we used descriptive statistics (frequencies and percentages) to present study parameters. Quality assessment of the included publications was not conducted.

**Table 2:** Key characteristics of included articles.

Reference	Location	Type	Theoretical Lens	Design	Methods	Sample Size
Lin, 2022 [26]	Canada	Acceptability	Equity lens	Cross-sectional	National survey	n = 3522, Migrants n = 598
Longchamps, et al., 2021 [24]	France	Acceptability	none reported	Cross-sectional	Questionnaire	n = 235, Migrants n = 217
Khaled et al., 2021 [21]	Qatar	Acceptability	none reported	Cross-sectional	National survey	n = 1038, Migrants n = 867 (White-collar n = 689, Blue-collar migrants n = 178)
Alabdulla et al., 2021 [18]	Qatar	Acceptability	none reported	Cross-sectional	National survey	n = 7,821, Migrants n = 5,925
Robertson et al., 2021 [25]	UK	Acceptability	none reported	Cross-sectional	National survey	n = 12,035, n = 9,981 (weighted sample), From weighted sample Migrants n = 824 (Not Born in UK)
Aktürk et al, 2021 [19]	Germany	Acceptability & Uptake	none reported	Cross-sectional	Questionnaires	n = 420, Migrants n = 348 (migratory background)
Deal et al., 2021 [20]	UK	Acceptability & Access	The ‘Three Cs’ model	Qualitative	Semi-structured interviews	n = 32 recently arrived migrants
West et al., 2021 [27]	India	Acceptability	WHO Behavioral and Social Drivers of vaccination (BeSD) model	Observational	Survey	n = 360 Temporary foreign workers (TFWs)
Knights et al., 2021 [22]	UK	Access, Acceptability	none reported	Qualitative	Semi-structured interviews	n = 81, Migrants n = 17
Iacoella et al., 2021 [23]	Italy	Acceptability	none reported	Cross-sectional	Survey	n = 112, Migrants n = 78

## Results

### Characteristics of included articles

**Fig 1:**
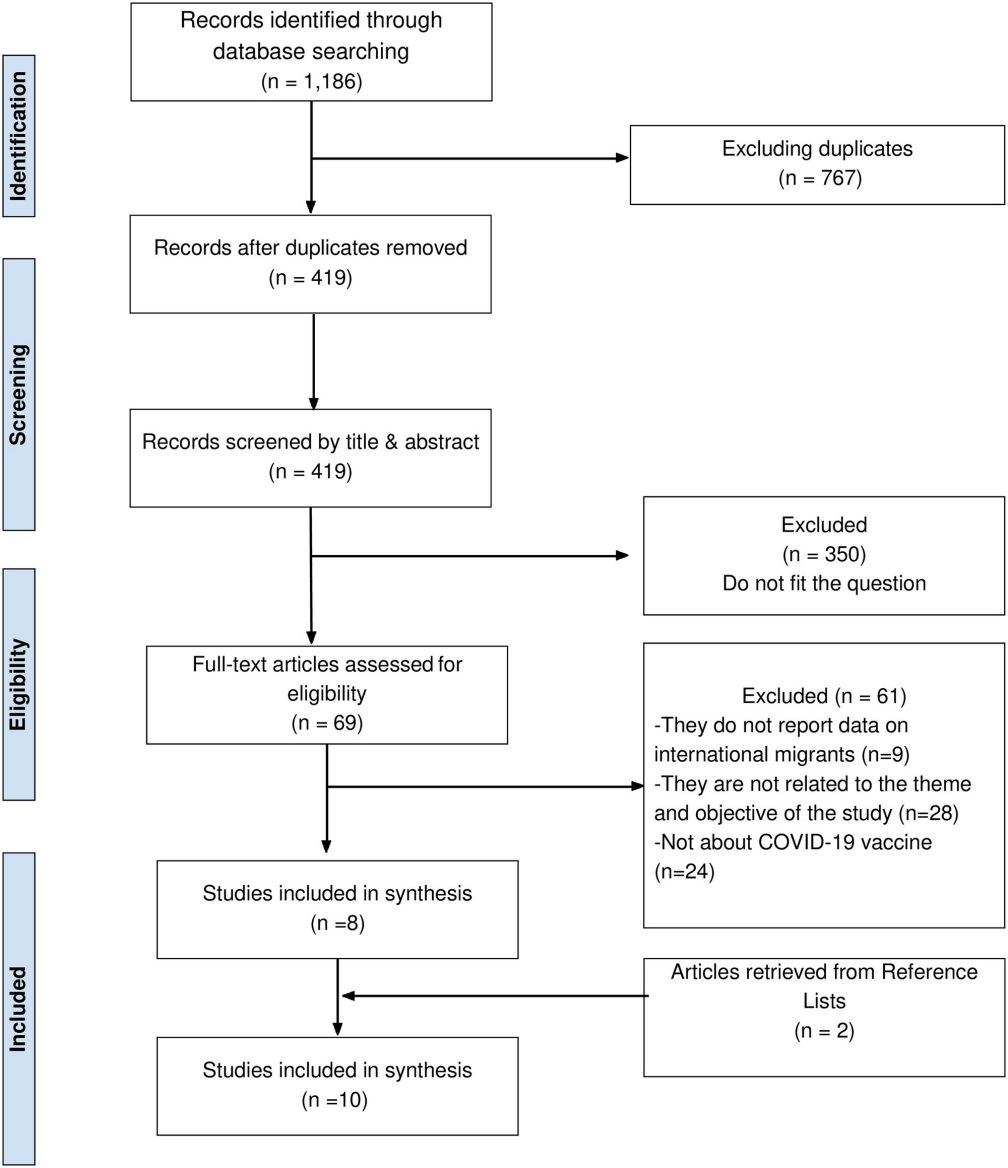


As shown in Fig 1, the systematic search yielded 1, 186 records. After duplicates were removed, 419 records were screened based on the titles and abstracts; 350 records were excluded. The full texts of 69 articles were screened; eight met the eligibility criteria, as they focused on the access, acceptability, and/or uptake of the COVID-19 vaccine among global migrants. Two more articles were identified in the reference lists of the included articles; 10 articles were included for synthesis in this rapid review.

Of the analyzed articles, all authors reported data on the acceptability of the COVID-19 vaccine [18–27]. Two studies covered access [20–22] and one article reported on the uptake of vaccines [18]. Eight articles followed a quantitative approach, and two studies used qualitative methods [20–22]; only three reported using a theoretical lens: equity [26], behavioral and social drivers for vaccination [27], and the three C’s model. [20]. Table 2 summarizes the key characteristics of the included articles, and Table 3 summarizes the key findings of the included articles.

**Table 3:** Key findings of included articles.

Reference	Key Findings
	**Access & Acceptability**
Deal et al., 2021 [20]	72% of migrants reported being hesitant about accepting a COVID-19 vaccine and (6%) would not accept the vaccine. Convenience of access was considered a key determinant around vaccine acceptability.
Knights et al., 2021 [22]	Difficulties in digital literacy and access to technology in addition to language barriers have made it challenging for migrants to access Covid-19 information and interventions.
	**Uptake & Acceptability**
Akturk et al, 2021 [19]	42.3% of migrants considered getting vaccinated, this proportion was 76.5% for non-immigrant Germans. 4.6% (n = 16) had already received the COVID-19 vaccine. Positive reasons to get vaccinated: for self-protection (16.7%) and vaccination is the effective solution (6.8%). · Negative reasons to get vaccinated: safety concerns (15.3%) and vaccines too rapidly developed (9.5%).
	**Acceptability**
Lin, 2022 [26]	Migrants reported higher concerns for vaccine acceptability when compared to Canadians, vaccine safety (71.3% vs. 49.5%), side effects (66.4% vs 47.3%) and mistrust in vaccinations (12.5% vs 6.6%). Prevalence of vaccine hesitancy (16.9%) in migrants was higher than Canadians (21.5% vs. 15.5%, p<0.001). Migrants had higher odds-on risk perception of accessing care (aOR 2.44, 95% CI 1.89–3.15), anticipated stigma of being targeted (aOR 2.24, 95% CI 1.81, 2.78) and COVID-19 vaccine hesitancy (aOR 1.99, 95% CI 1.57–2.52)
Longchamps, et al., 2021 [24]	40.9% of migrants reported not being willing to get vaccinated against COVID-19.
Khaled et al., 2021 [21]	42.7% of migrants were accepting the COVID-19 vaccine, 45.2% were hesitant, and 12.1% resistant.
Alabdulla et al., 2021 [18]	20.2% of migrants were not willing to be vaccinated, and 19.8% were unsure about being vaccinated. Migrants had lower hesitancy (16.71%) compared to Qataris of working age (42.57%) to get the COVID-19 vaccine.
Robertson et al., 2021 [25]	There was no difference in vaccine hesitancy in migrants and non-migrants (OR 0.99 95% CI: 0.67, 1.48).
West et al., 2021 [27]	Vaccine hesitancy was 25% among migrants. Undocumented migrants had higher levels of vaccine hesitancy. 41%) compared to those with valid visas (22%, p = .009)
Iacoella et al., 2021 [23]	64.3% of migrants were willing to get vaccinated, 3.6% were not sure, and 32.1% did not want to receive the vaccine.

### Findings related to COVID-19 vaccine access

Twenty percent (n = 2) of the included articles examined COVID-19 vaccine access among global migrants [20, 22]. The average age, 37.55 years of participants in these studies represented that of working-age migrants. Female participants represented most of the samples in both research articles: 66% [19] and 64.7% [22] respectively. Migrants originated primarily from countries in Africa, the Eastern Mediterranean, and Southeast Asia. According to these articles’ authors, global migrants face specific challenges to vaccine access. Technological barriers prevent migrants from accessing vaccine information and application [22]. Not owning technology, not knowing how to use it appropriately, and being unable to maintain it are some of the barriers found [22]. In some cases, migrants—particularly undocumented migrants—assumed that they did not have access to the vaccine. The undocumented migrants believed that their immigration status was an issue [20].

### Findings related to COVID-19 vaccine acceptability

All the studies included in this review assessed COVID-19 vaccine acceptability. Most articles (70%) reported that male participants predominated their samples, and the majority were of working age (18–50 years); no study included children or adolescent populations. Some articles targeted specific types of migrants. For instance, research conducted in France [24] and Italy [23] focused on migrants who were experiencing homelessness. Other research focused on economic migrants in Qatar [18] and temporary foreign workers in India [27]. Participants were mainly from Africa, Eastern Mediterranean, Asia, and South America.

According to the studies included in this rapid review, most global migrants are hesitant to get vaccinated for COVID-19 [18–22, 24–27]; this is different from one study where more than 60% of migrants were willing to get the vaccine [23]. The top concerns related to hesitancy and acceptability include mistrust in vaccinations and in the health system [19, 20, 26], presenting vaccine side effects [20, 26], vaccine safety [26], and believing that the vaccine is understudied [19]. Undocumented migrants had additional concerns, including the fear of encountering immigration checks [20]. A key determinant of vaccine acceptability is convenient access to the vaccine [20]. Similarly, perceiving the vaccine as a source of self-protection and as an effective solution to COVID-19 were reasons to accept the vaccine [19]. The key findings of the included articles are summarized in Table 3.

### Findings related to COVID-19 vaccine uptake

One article explored the uptake of the COVID-19 vaccine [19]. Most of the participants were male (62.1%), and the average age was 39.3 years, indicating that the study participants were of working age [19]. The migrants’ countries of origin were Turkey (91%) and Bulgaria (9%) [19]. In this study, less than 50% of the migrants considered getting vaccinated, and 4.6% had already received the vaccine [19].

## Discussion

This rapid review aimed at synthesizing evidence on COVID-19 vaccine access, acceptance, and uptake among global migrants.

Most studies used quantitative methodologies. This may be due to the methodological strengths of quantitative research designs to generalize findings to the broader population for public health interventions. However, we argue that global migrants come from diverse backgrounds with complex experiences prior to, during, and after their migration processes, which may shape their everyday lives and decisions, including COVID-19 vaccination [27–29]. Thus, quantitative designs may not fully capture all the details behind COVID-19 vaccine access, acceptability, and uptake among global migrants, warranting the need to adopt qualitative and mixed methods approaches for a more contextualized understanding of the COVID-19 vaccination among this population.

We found low acceptability and high hesitancy toward COVID-19 vaccines among global migrants across most of the studies except for the Italian study [23]. These findings corroborate past evidence that showed a higher risk of under-immunization for routine and seasonal vaccines among migrants when compared with the general population before the COVID-19 pandemic [28, 29]. Similarly, the cited reasons for the low acceptance and uptake of the COVID-19 vaccine were not different from contributing factors of vaccine hesitancy toward routine vaccines among global migrants; they included trust issues with vaccines and health systems, as well as safety concerns [30]. Precarious migration status and concerns about COVID-19 vaccine development and research contributed to a lower acceptance and uptake of the COVID-19 vaccine [30]. Although COVID-19 vaccination programs in some jurisdictions were independent of immigration and citizenship statuses, the collection of demographic information for statistics and record keeping might have dissuaded some undocumented migrants from receiving the vaccines, as they feared that they may be tracked and repatriated [31]. The higher acceptance of COVID-19 vaccines in the Italian study may be due to most participants migrating from the neighboring European Union Economic Zone countries and having a similar cultural, health system, and economic background as Italians, which alleviated concerns for hesitancy [23]. European migrants in Italy might not be experiencing the immigration and trust issues cited in the other studies. Multiple intersecting factors may influence vaccine acceptance and hesitancy [32, 33]. In this regard, previous work suggested that considering diverse and inclusive approaches to vaccination, such as clear information and communication, and community engagement strengthens vaccine confidence and acceptance among global migrants [34]. Furthermore, geopolitical, and international economic relations between migrants’ countries of origin and the host country may be a modifying factor for vaccine acceptance and uptake among migrants. Future research may be conducted to investigate our assumption.

The COVID-19 pandemic and vaccination are evolving. Hence, communication and information on COVID-19 vaccination in host nations for global migrants should be a continuous practice—one tailored at meeting their linguistic and diverse changing needs. This can be achieved by involving global migrants at various levels of COVID-19 vaccination communication programs and ensuring that information is easily culturally accessible, evidence informed, and relevant for enhancing trust and confidence. Trust and confidence are key contributory factors to COVID-19 vaccination acceptability and uptake among global migrants. In this regard, we suggest that more qualitative exploration will offer a nuanced understanding of this topic to generate contextual insights that may shape COVID-19 vaccination practices among global migrants.

### Strengths and limitations

In this rapid review, we synthesized up-to-date information on COVID-19 access, acceptability, and uptake among global migrants. The strengths of our study lie in the meticulousness with which we conducted the search, selection, and analysis of articles, as well as our inclusion of publications in multiple languages. However, it is important to note that our review was limited to articles published in specific languages and databases. As a result, the ability to draw comprehensive conclusions may be somewhat constrained due to the exclusion of reports from other languages and databases.. There is a lack of articles that adequately represent the perspectives and viewpoints from specific countries. Still, the evidence synthesized in our review originated from major host destination countries for global migrants according to the World Migration Report [4] and others [35] which strengthened the quality and relevance of our findings. The review encompassed articles with small sample sizes, which may have restricted statistical power and limited ability to yield results that can be generalized. Consequently, it is crucial to exercise caution when interpreting the findings from these studies and acknowledge their limitations in terms of generalizability. The review had a small subset of countries; thus, the findings may not accurately represent the global or regional variations in terms of cultural, socioeconomic, and geopolitical factors that influence the access, acceptability and uptake of the COVID-19 vaccine among global migrants.

## Implications and conclusion

This rapid review provides an overview of the access, acceptability, and uptake of the COVID-19 vaccine. Our findings suggest that quantitative approaches prevail in this area of research. We recommend that researchers consider further expanding the knowledge and understanding of the contributors to vaccine hesitancy among migrant populations by using research designs and methods that capture the nuances of experiences (e.g., distress, precarity, and mental health problems) and how intersections of identities (e.g., age, gender, sex, language, nationality, and race) and social locations (e.g., class, income, employment, and residential status) influence vaccination attitudes and decisions among global migrants. Examining vaccination through an intersectional lens can be beneficial for uncovering underlying interactions among social locations, power inequalities, and systems of oppression that produce specific barriers to vaccination. Involving stakeholders and key informants in research can generate culturally appropriate interventions for increasing the access, acceptability, and uptake of the COVID-19 vaccine and others among global migrants. The findings from published studies confirm low acceptability and high hesitancy in vaccination among global migrants, especially those with undocumented status. In this regard, we recommend both immunization program focus and individual patient focus approaches to address vaccine hesitancy and to improve vaccine access, acceptability, and uptake. These tailored interventions can be guided by scientifically-proven methods such as motivational interviewing, a client-centered method designed to help people to find the motivation to make positive health decisions, particularly immunization.
